# Osteoarthritis treatment with a novel nutraceutical acetylated ligstroside aglycone, a chemically modified extra-virgin olive oil polyphenol

**DOI:** 10.1177/2041731420922701

**Published:** 2020-05-27

**Authors:** María C de Andrés, Mia S Meiss, Marina Sánchez-Hidalgo, Alejandro González-Benjumea, Jose G Fernández-Bolaños, Catalina Alarcón-de-la-Lastra, Richard OC Oreffo

**Affiliations:** 1 Bone and Joint Research Group, Centre for Human Development Stem Cells and Regeneration, Institute of Developmental Science, University of Southampton, Southampton, UK; 2 Cartilage Epigenetics Group, Rheumatology Division, INIBIC-Complexo Hospitalario Universitario A Coruña (CHUAC), A Coruña, Spain; 3 Department of Pharmacology, Faculty of Pharmacy, University of Seville, Sevilla, Spain; 4 Department of Organic Chemistry, Faculty of Chemistry, University of Seville, Sevilla, Spain

**Keywords:** Cartilage, osteoarthritis, nutraceuticals

## Abstract

Recent studies have shown that dietary patterns confer protection from certain chronic diseases related to oxidative stress, the immune system and chronic low-grade inflammatory diseases. The aim of this study was to evaluate the anti-inflammatory potential and the capacity to attenuate cartilage degradation using extra-virgin olive oil–derived polyphenols for the treatment of osteoarthritis. Results show that both nutraceuticals ligstroside aglycone and acetylated ligstroside aglycone showed an anti-inflammatory profile. Acetylated ligstroside aglycone significantly reduced the expression of pro-inflammatory genes including NOS2 and MMP13 at both RNA and protein levels; decreased nitric oxide release; and, importantly, reduced proteoglycan loss in human osteoarthritis cartilage explants. Our study demonstrated that a new synthetic acetylated ligstroside aglycone derivative offers enhanced anti-inflammatory profile than the natural nutraceutical compound in osteoarthritis. These results substantiate the role of nutraceuticals in osteoarthritis with implications for therapeutic intervention and our understanding of osteoarthritis pathophysiology.

## Introduction

Osteoarthritis (OA) remains, currently, the most frequent cause of pain, deformity and dysfunction in the elderly.^1^ OA is a late-onset, complex disease of the joint, characterised by progressive failure of the extracellular cartilage matrix (ECM), together with changes in the synovium and subchondral bone. OA persists as the most common form of arthritis worldwide and the sixth leading cause of disability.^2^ Unlike most tissues, articular cartilage does not contain blood vessels, nerves or lymphatics, rather, articular cartilage is composed of a dense ECM with a sparse distribution of highly specialised cells called chondrocytes.^3^ Aberrant expression of degradative proteases or catabolic mediators is induced in OA chondrocytes that contributes to cartilage destruction.^4^ Articular cartilage repair and regeneration remain largely intractable due to the poor regenerative properties of this tissue. A variety of different promising strategies incorporating stem cells, biomaterials and growth factors among others are currently being examined in order to regenerate this unique tissue.^5–7^

To date, there is no definitive cure for this debilitating disease. The mechanism of disease progression in OA remains largely unknown and thus, to date, a more personalised approach is required to aid patient disease management. Current treatments are targeted at reducing symptoms of the inflammatory reaction that occurs following destruction of the essential joint cartilage. These treatments, however, do not prevent the significant pain associated with OA or the often reported restriction of mobility and activity.

Recent studies have shown that dietary patterns, including the traditional Mediterranean diet, characterised by enhanced levels of naturally derived vegetables, fruits, nuts, fish/fish oils and grains, confer protection from certain chronic diseases related to oxidative stress, the immune system and chronic low-grade inflammatory diseases.^8^ Recently, data from the Osteoarthritis Initiative (OAI) have demonstrated that adherence to the Mediterranean diet is associated not only with better quality of life^9^ but also, significantly, with a lower prevalence of OA.^10^ Given that the general population can be viewed as at risk in the development of OA in later life, an approach that relies on dietary modification is attractive in terms of risk/benefit and, potentially, an approach that is more likely to be implementable.^11^ Indeed, as an alternative to traditional treatments, alternative modalities have come to the fore including the effects of nutraceuticals as non-invasive treatments, based on the evidence that epigenetic changes are triggered by dietary nutrients and contribute to the prevention of a number of diseases.^12^ ‘Nutraceutical’ is a substance that may be considered a food or part of a food, which provides medical or health benefits, encompassing prevention and treatment of disease.^13^ A number of compounds present in the diet (vitamins, carotenoids, flavonoids) have been shown to modulate activity in laboratory models of OA and/or the human disease,^11^ including the effect of vitamin D supplementation on articular cartilage morphology.^14^ Many of these compounds are inhibitors of the nuclear transcription factor-κB (NF-κB) pathway.^15^ Although initially considered a cartilaginous disease, OA displays a much more complex pathology with inflammatory mediators released by bone, cartilage and synovium. Low-grade inflammation induced by metabolic syndrome, innate immunity and chronic inflammation (‘inflammaging’) are some of the more recent arguments in favour of an inflammatory role in OA^16^ and, for the link between inflammation and epigenetic regulation in OA.^17^ Thus, there is an increasing interest in the potential of nutraceuticals, not only for treatment of musculoskeletal diseases^18–21^ but also as alternative approaches to be evaluated in cancer^22,23^ and sclerosis,^24^ among others.

The Mediterranean diet contains extra-virgin olive oil (EVOO) as the main lipid source resulting in a high intake of phytonutrients including vitamins and natural phenols. These phenols are secondary plant metabolites that are characterised chemically by having one or more aromatic rings with one or more hydroxyl substituents. The phenols are present in the lipid and water from the olive tree (*Olea europea L.*) and, fractions of EVOO, and display remarkable properties including antioxidant, anticarcinogenic, anti-inflammatory and immunomodulatory actions.^12^

A relevant polyphenol in EVOO is ligstroside aglycone (LA) (also called p-HPEA-Elenolic acid).^25^ While information on LA bioactivity is limited, a few years ago, LA was demonstrated to behave as an antioxidant^26^; furthermore, LA has been shown to have anti-inflammatory effects by controlling and downregulating NF-κB as well as the potential to induce a caloric restriction-like state that affects the muscle, brain, fat tissue and kidney, particularly through activation and increased levels of sirtuins.^12^

The current study has examined the effects of LA and a chemically acetylated version of LA: acetylated ligstroside aglycone (A-LA) in comparison to the natural polyphenol as a potential treatment for OA. Acylation of phenolic compounds may confer beneficial properties to these molecules, including cell membrane penetration, improved bioavailability, enhanced antitumor, anti-inflammatory and antimicrobial activities.^27,28^ This new LA derivative may offer a new promising therapeutic strategy in the management of inflammatory-related pathologies.

## Methods

### Cartilage dissection and chondrocytes isolation

Human OA articular cartilage was obtained from six patients following total hip arthroplasty (1 male and 5 female patients with a mean ± SD age of 60 ± 13.8 years). Informed consent was obtained from all patients and the study was approved by the Southampton & South West Hampshire Local Research Ethics Committee (LREC 210/01). Full thickness cartilage was dissected from the femoral heads and cut into punches using a 4 mm biopsy punch. Punches were allowed to equilibrate overnight in DMEM/F12 (Life Technologies) supplemented with 5% foetal calf serum (FCS; Invitrogen), 1% insulin–transferrin–selenium (ITS; Sigma–Aldrich), 100 units/mL of penicillin, 100 μg/mL of streptomycin (P/S; Lonza) and 100 μg/mL of ascorbic acid (Sigma–Aldrich), referred to as basal media, in 5% CO_2_ at 37°C. Only chondrocytes from the superficial layer of OA cartilage were isolated as previously described.^29,30^

### Tissue culture

The cartilage punches were stimulated on days 0, 3, 7, 10, 14. Each patient had three punches per condition: (1) basal media, (2) 5 ng/mL IL-1β and 5 ng/mL oncostatin-M (OSM) (IL-1β/OSM), (3) IL-1β/OSM and 10 μM LA, (4) IL-1β/OSM and 50 μM LA, (5) IL-1β/OSM and 10 μM A-LA and (6) IL-1β/OSM and 50 μM A-LA.

At days 7 and 14, a punch was removed from each condition and fixed overnight in freshly prepared paraformaldehyde, dehydrated in ethanol washes and incubated in histoclear prior to embedding in paraffin wax for further immunohistochemical analysis. The final punch was flash frozen in liquid nitrogen after day 14 and kept at –80°C for DNA/RNA extraction.

### Cell culture

Chondrocytes were cultured at a density of 1–3 × 10^6^ cells/25 cm^2^ flask in basal media at 5% CO_2_ at 37°C until confluent. Cells were split and plated in 24 well plates to provide a density of 50,000 cells/well for 6 and 24 h analysis. Cells were left to equilibrate overnight in basal media in 5% CO_2_ at 37°C. Cells were then stimulated as for the cartilage punches. After 6 and 24 h, respectively, the supernatant was collected and frozen at –80°C. The cells were lysed using Buffer RLT Plus (Qiagen) and kept at –80°C for later analysis.

### Griess assay for nitrite analysis

The Griess Reagent system (Promega) was used to quantify the nitrite levels in the supernatants taken at 24 and 48 h, day 3, 7, 10 and 14 using sodium nitrite, as the standard. Briefly, 50 μL of culture supernatant was incubated with 50 μL of 1% sulfanilamide for 10 min and then, with 0.1% N-1-naphthylethylenediamide dihydrochloride solution at room temperature for 5 min. The optical density was then measured at 555 nm.

### Sulfated glycosaminoglycan assay

The release of proteoglycan (PG) was determined using a spectrophotometric assay, as sulfated glycosaminoglycan (s-GAG) according to manufacturer’s instructions (Biocolor). Briefly, the reaction between s-GAG and dye, produced a dye-GAG complex that precipitated out within 30 min. Then, s-GAG-bound dye was recovered by adding 1 mL of dissociation reagent. Finally, the s-GAG content of the assayed samples was determined by the amount of dye recovered from the s-GAGs in the test sample by measuring absorbance at 656 nm and was expressed as ng GAG/mg weight of cartilage.

### Histological analysis

Cartilage punches were divided in two to reveal in cross section the superficial to deep zone such that after embedding cartilage samples were sectioned at 6 μm thickness to show all layers of cartilage. Following slide de-waxing and rehydration, samples were treated with haematoxylin and stained with either Alcian blue/Sirius red or Safranin-O dyes. Samples were subsequently imaged using a Zeiss Universal light microscope (Zeiss) and images were captured with a digital camera.

### Immunohistochemistry

Formalin-fixed paraffin-embedded cartilage sections were deparaffinised and permeabilised in 0.5% Triton X-100 (Sigma-Aldrich) for 1 h. Any endogenous peroxidase activity was blocked by incubating the slides in 3% H_2_O_2_ for 5 min. Samples were incubated in blocking buffer (1% bovine serum albumin (BSA) in phosphate buffered saline (PBS)) for 5 min and incubated overnight at 4°C in anti-NOS2 (BML-SA200; Enzo Life Sciences International) diluted 1:125 with 1% BSA–PBS or rabbit anti-human matrix metalloproteinase 13 (MMP-13) (AHP751; Serotec) diluted 1:50. The antibody was visualised using the appropriate biotinylated secondary antibody, followed by treatment with avidin–peroxidase and 3-amino-9-ethyl-carbazole. Sections were counterstained with 1% Alcian blue and viewed with a Zeiss Universal light microscope (Zeiss), followed by image capture with a digital camera. Positively stained cells were quantified using software ImageJ 1.51j8.

### DNA and RNA extraction

OA cartilage punches from day 14 were homogenised in Buffer RLT Plus and total RNA and genomic DNA extracted simultaneously from digested samples using an AllPrep DNA/RNA/miRNA Universal kit (Qiagen), according to the manufacturer’s instructions. Due to low levels of RNA, it was immediately reverse transcribed with SuperScript VILO cDNA Synthesis Kit (Life Technologies). The cell lysates were also processed using the same kit. RNA was immediately reverse transcribed with TaqMan Reverse Transcription Reagents (Applied Biosystems).

### Quantitative reverse transcription polymerase chain reaction (qRT-PRC)

Relative quantification of gene expression was performed with an ABI Prism 7500 detection system (Applied Biosystems). A 20 μL reaction mixture was prepared in duplicate, containing 1 μL of complementary DNA, 10 μL of GoTaq (Promega), 5 μL upH_2_O and 2 μL of forward primer and 2 μL reverse primer of gene of interest. The final mixture of 20 μL was then added to a 96 well-plate and thermal cycler conditions included an initial activation step at 95°C for 10 min, followed by a two-step polymerase chain reaction (PCR) programme of 95°C for 15 s and 60°C for 60 s for 40 cycles. *ACTB*, an endogenous housekeeping gene was used to normalise Ct (cross-over threshold) values using the 2^−ΔΔCt^ method for relative quantification of gene expression. All reactions were performed in duplicate and included a negative control with no cDNA (primer information upon request).

### Measurement of cytokines in supernatants by Luminex technology

The concentrations of IL-8, IL-1β, MMP-13, TNFα, Tissue Inhibitor of Metalloproteinase-1 (TIMP-1) and aggrecan were measured in supernatants harvested at day 14. The assays were undertaken using human magnetic Luminex multiplex kits in accordance to the manufacturer’s instructions (R&D Systems). The plates were read on a Bio-plex 200 System. The assay sensitivity for each analyte was IL-8: 1.8 pg/mL, IL-1β: 0.8 pg/mL, MMP-13: 19.0 pg/mL, TNFα: 1.2 pg/mL, TIMP-1: 3.42 pg/mL and aggrecan: 249 pg/mL.

### Statistical analysis

Statistical analysis was performed using SPSS software version 21. Except where indicated otherwise, data are presented as the mean ± SEM of at least three multiple independent experiments. Significance was determined by analysis of variance with post hoc t-test and by Mann–Whitney U test; *p* values less than 0.05 were considered significant.

## Results

### A-LA inhibited nitrite production and S-GAG release in cytokine stimulated OA cartilage

**Figure 1.**
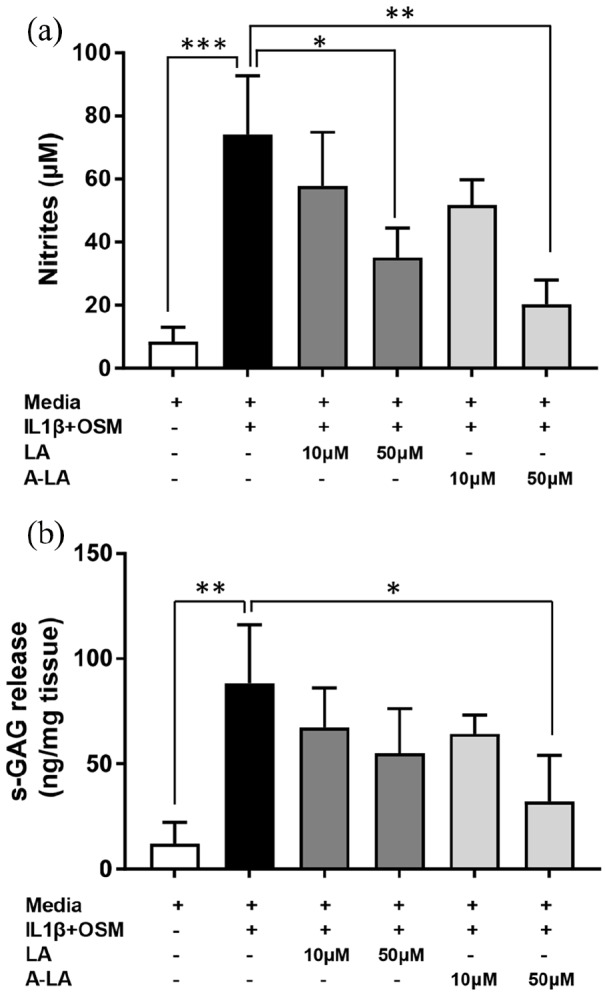
Effects of LA and A-LA on IL-1β + OSM-induced NO production (a) and s-GAG release (b). Cartilage explants were stimulated for 14 days, supernatants collected and NO and s-GAG concentrations were determined by colorimetric reactions. For PG loss, results are expressed as ng s-GAG per mg tissue. Data represent mean ± SEM (n = 6). *p ⩽ 0.05, **p ⩽ 0.01, *p ⩽ 0.001 versus IL-1β + OSM group.

Human articular cartilage was dissected from the femoral heads of OA patients undergoing total hip arthroplasty and cartilage punches stimulated by culturing in the nutraceuticals LA and A-LA at 10 and 50 μM for 14 days. Supernatants collected from the stimulated cartilage punches throughout the culture period (24 h to 14 days) were assayed for nitrite (NO_2_–) content using the Griess assay. The success of the inflammatory OA model, used as a positive control, was evident following stimulation of the cartilage punches with IL-1β and OSM compared to basal media punches with significantly increased nitrite measurements observed (74.03 ± 18.70 vs 8.43 ± 4.57; *p* = 0.001) (Figure 1(a)). The highest nitrite levels were noted after 7 days in all conditions examined (data not shown). After 48 h post stimulation, both compounds and concentrations reduced nitrite levels compared to the positive control, with enhanced reductions observed at higher concentrations. The results showed that only 50 μM A-LA reduced nitrite levels from day 3 onwards, with the greatest significant difference observed at day 14 (20.33 ± 7.59 vs 74.03 ± 18.70; *p* = 0.004). In contrast, 50 μM LA treatment (35.14 ± 9.34 vs 74.03 ± 18.70; *p* = 0.030) only reduced significantly the levels of nitrite at day 14 (Figure 1(a)).

PG release, measured by s-GAG levels, was assessed in supernatants from day 14 of tissue culture of OA cartilage punches exposed to the tested compounds (Figure 1(b)). Stimulation with cytokines IL-1β and OSM significantly increased the amount of s-GAG released per mg tissue (88.49 ± 27.58 vs 12.06 ± 10.29; *p* = 0.009). In contrast, treatment of the cartilage punches with 50 μM of both compounds reduced the levels of s-GAG release into the supernatants, an effect that followed a dose-dependent trend, although no conditions examined reduced PG loss to the levels observed in the control (Figure 1(b)). Statistical significance was only detected following treatment with 50 μM A-LA (32.11 ± 21.94 vs 88.49 ± 27.58; *p* = 0.047) (Figure 1(b)).

### Reduction in PG loss from OA cartilage following treatment with nutraceuticals

**Figure 2.**
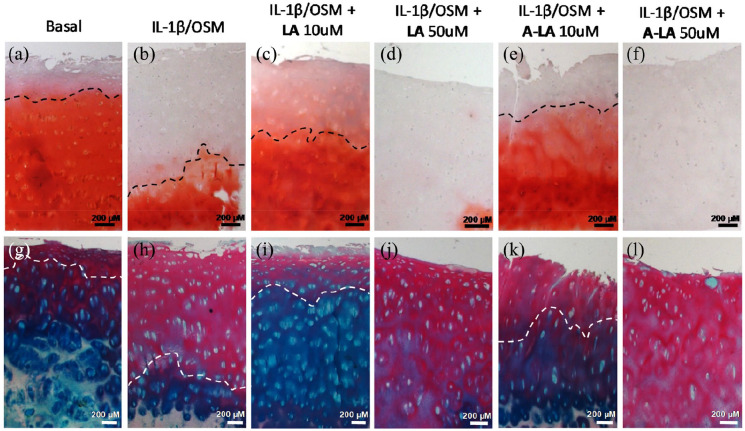
Stimulation with A-LA recovered PG losses. OA cartilage punches were cultured for 14 days in different conditions then sectioned to show the top superficial layer down to the deep zone at the bottom. (a–g) Control group, (b–h) IL-1β + OSM group, (c–i) IL-1β + OSM + LA 10 µM group, (d–j) IL-1β + OSM + LA 50 µM group, (e–k) IL-1β + OSM + A-LA 10 µM group, (f–l) IL-1β + OSM + A-LA 50 µM group. Top row show Safranin-O staining for PG and bottom row Alcian blue/Sirius red for PG and collagen in matrix, respectively. All scale bars represent 200 µm.

To further analyse the effects on PG loss, OA cartilage punches were sectioned to evaluate PG expression across the cartilage cross-sections (superficial to deep zone) following exposure to nutraceuticals for 14 days. PG was visualised in the cartilage matrix following staining with Safranin-O and Fast Green, as a counterstain (Figure 2(a)–(f)). The positive control cartilage punch samples, exposed to the inflammatory cytokines IL-1β and OSM in media, displayed the greatest reduction in PG in the cartilage, with only the deep zone layers stained red (Figure 2(b)).

Treatment with both 10 μM LA (Figure 2(c)) and 10 μM A-LA (Figure 2(e)) recovered the loss in PG induced by IL-1β and OSM with evidence of PG staining across the cartilage up to the superficial layer, almost comparable to that observed for cartilage punches maintained in basal media (Figure 2(a)); particularly evident in the A-LA treatment group. However, culture using 50 μM LA (Figure 2(d)) and 50 μM A-LA (Figure 2(f)) depleted PG expression in the ECM with negligible staining visible, an observation consistently observed across all patients. Similarly, staining with Alcian Blue, and counterstain with Sirius Red (Figure 2(g)–(l)), showed that 10 μM of the tested compounds recovered PG losses (Figure 2(i) and (k)). Again, treatment with 50 μM of both compounds (Figure 2(j) and (l)) exacerbated the loss observed in the positive control group (Figure 2(h)).

Given the deleterious effects following exposure to 50 μM of any of the nutraceuticals and, the similarly negative effects observed in cell culture using chondrocytes isolated from OA cartilage from patients (data not shown), all further analysis focussed on samples exposed to 10 μM of the EVOO compounds.

### Reduction of positive staining for NOS2 and MMP-13 in OA cartilage after stimulation with A-LA

**Figure 3.**
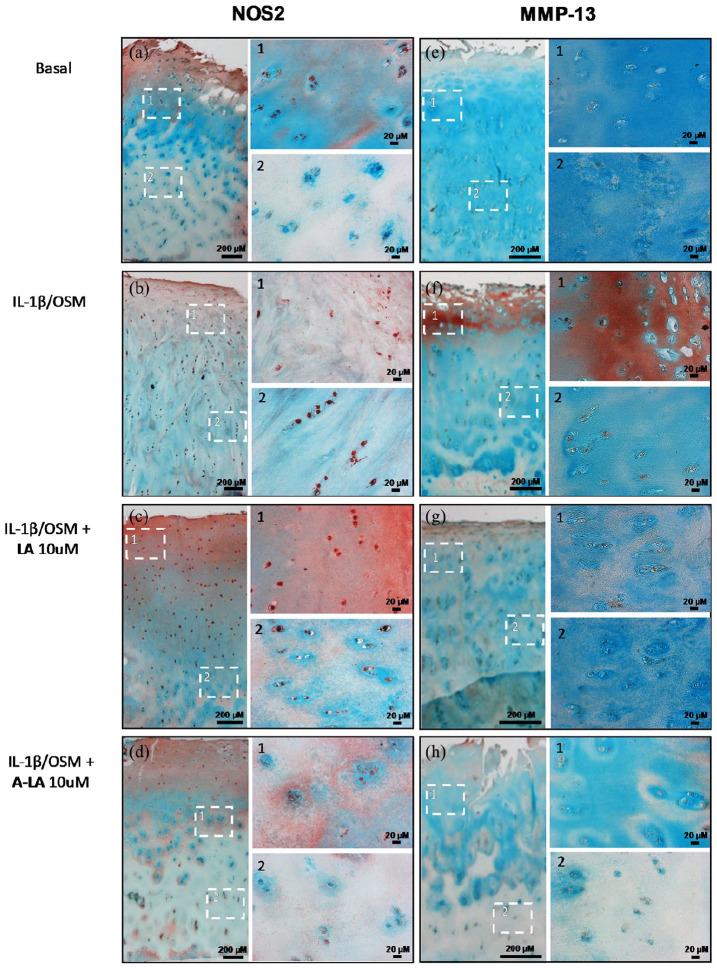
Treatment with A-LA reduced positive staining for catabolic mediators in cartilage explants. Punches from OA cartilage were cultured for 14 days in different conditions then cross-sectioned to show superficial on top with deep zone on bottom. (a–e) Control group, (b–f) IL-1β + OSM group, (c–g) IL-1β + OSM + LA 10 µM group, (d–h) IL-1β + OSM + A-LA 10 µM group, left column represents NOS2 staining and right column MMP-13 staining. Extracellular matrix was stained with alcian blue and light green. Images furthest left were taken at ×2.5 magnification while images 1 and 2 are zoomed in to framed areas with ×20 magnification. Unless otherwise indicated, scale bars are 200 µm. Shown are the representative images from n = 3.

Following exposure to 10 μM of the selected nutraceuticals for 14 days, NOS2 and MMP-13 expressions were determined by immunocytochemistry together with Alcian Blue and Light Green to stain the ECM (Figure 3). Culture of the cartilage punches in basal media resulted in NOS2-positive staining restricted to the superficial layer (Figure 3(a1)) while chondrocytes in the deep zone failed to show significant staining (Figure 3(a2)). Exposure to the inflammatory environment, using IL-1β and OSM, resulted in strong positive staining throughout the cartilage in all zones (Figure 3(b)). Significantly, treatment of the cartilage explants with 10 μM A-LA reversed the expression pattern with positive staining only observed in the superficial chondrocyte layer (Figure 3(d1)) with predominantly negative cell expression in the deep zone (Figure 3(d2)). This effect was not observed following treatment with 10 μM LA (Figure 3(c)). The data were also shown quantitatively, normalised to 100 μm^2^ tissue by counting the positively stained chondrocytes with a reduction evident in A-LA (2.36 ± 0.4 compared to positive control 3.26) although this did not reach statistical significance (data not shown). Similar results were observed for MMP-13-positive cells in cartilage explants following treatment with both nutraceuticals. Culture in basal media resulted in weakly stained chondrocytes predominantly in the superficial zone (Figure 3(e1)) while the positive control samples displayed intensely stained positive cells across the whole superficial layer (Figure 3(f1)), indicating the presence of MMP-13-producing chondrocytes. As observed above, exposure to 10 μM A-LA reduced the positively stained cells in the deep zone (Figure 3(h2)) and removed the positive stain of the ECM in the superficial zone (Figure 3(h1)), and quantitatively (2.32 ± 0.37 vs 3.62 ± 0.25, *p* = 0.05) (data not shown).

### Stimulation with A-LA reduced gene expression of key OA genes in chondrocytes

**Figure 4.**
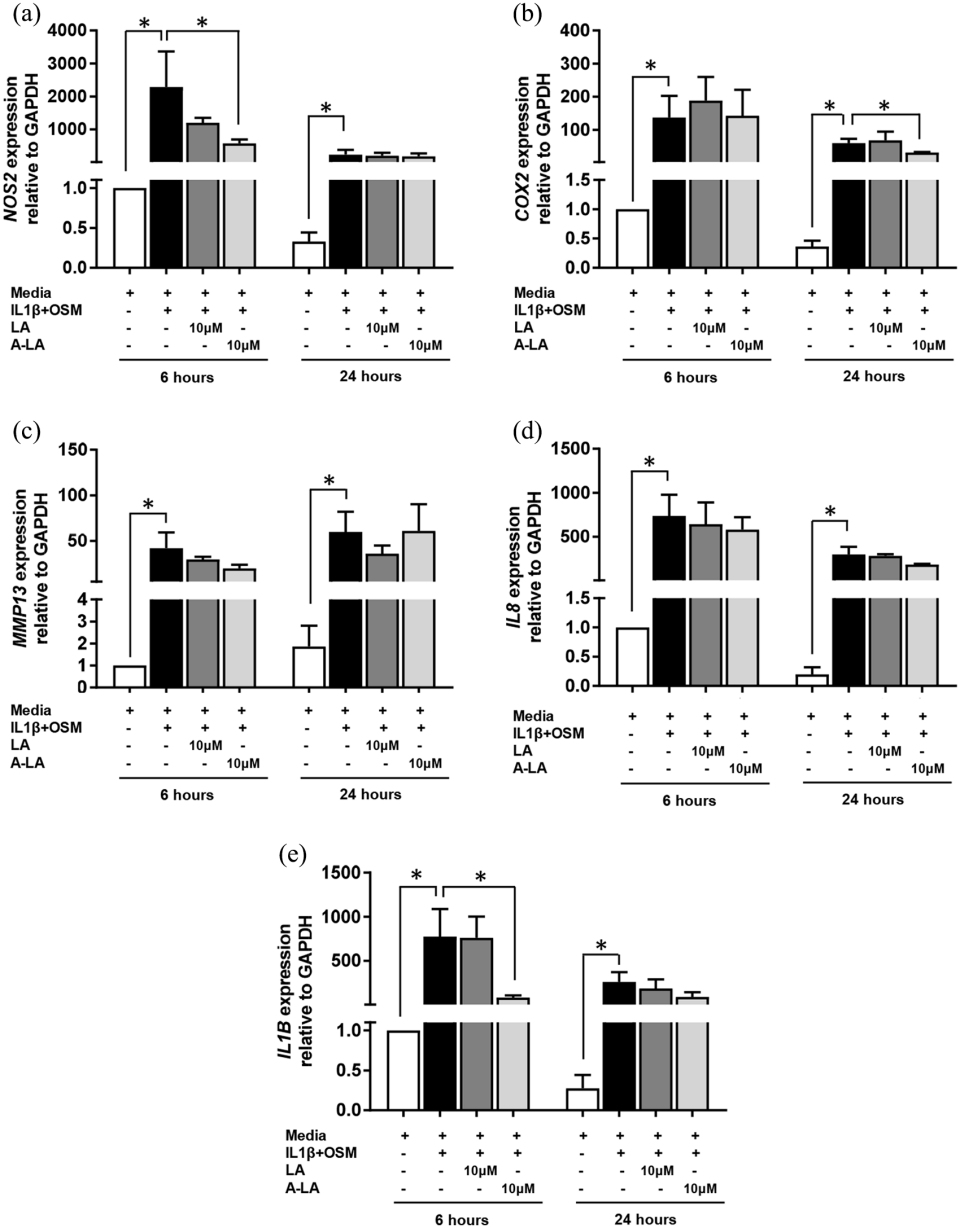
Reduced pro-inflammatory gene expression profile in OA chondrocytes after nutraceutical stimulation. (a) *NOS2*, (b) *COX2*, (c) *MMP13*, (d) *IL8* and (e) *IL1B*. Gene expression levels were measured from lysed chondrocytes after 6 and 24 h of culture with different conditions. Gene expression is relative to *ACTB* housekeeping gene. Results are shown as mean ± SEM of triplicate determinations per sample (n = 3). *p ⩽ 0.05 versus IL-1β + OSM group.

Chondrocytes were cultured with chosen nutraceuticals for 6 and 24 h before quantification of gene expression (Figure 4(a)–(e)). Culture with IL-1β and OSM, induced a significant increase in gene expression demonstrating the success of the OA model (e.g. at 6 h *NOS2* expression increased from 1.00 ± 0.00 to 2287.95 ± 1079.00, *p* = 0.037) (Figure 4(a)). In contrast, treatment with 10 μM A-LA reduced expression of *NOS2* significantly at 6 h (573.09 ± 216.91 vs 2287.95 ± 1859.05, *p* = 0.050) (Figure 4(a)); these results correspond with the reduction of positively stained cells observed for the NOS2 immunohistochemistry using the cartilage explants (Figure 3(a)–(d)). In contrast, culturing in A-LA did not affect cyclooxygenase-2 (*COX2*) gene expression until 24 h (30.95 ± 1.54 vs 59.96 ± 12.64, *p* = 0.050) with no decrease observed at 6 h (Figure 4(b)). *MMP13* expression was reduced at 6 h again corresponding to the results from immunohistochemistry although no statistical significance was detected (19.61 ± 4.01 vs 41.90 ± 2.96, *p* = 0.275) (Figure 4(c)). In addition, *IL8* expression was reduced although no statistical significance was detected (at 6 h: 580.50 ± 144.51 vs 735.6 ± 242.32 and, at 24 h: 183.33 ± 9.33 vs 298.02 ± 87.27, *p* = 0.513) (Figure 4(c)). *IL1B* expression was significantly reduced at 6 h (81.83 ± 23.38 vs 774.88 ± 312.00, *p* = 0.050) with similar reductions that those seen at 24 h although this was not statistically significant (89.14 ± 53.56 vs 259.77 ± 110.55, *p* = 0.275) (Figure 4(d)). RNA was also extracted from cartilage punches cultured for 14 days and no conclusive results were found; however, given the limited amounts of RNA that could be extracted due to the small size of each OA cartilage sample, this may explain the lack of a clear pattern in the results obtained from the qRT-PCR (data not shown).

### Chemokine and cytokine expression profiles vary in OA cartilage explants following A-LA stimulation

**Figure 5.**
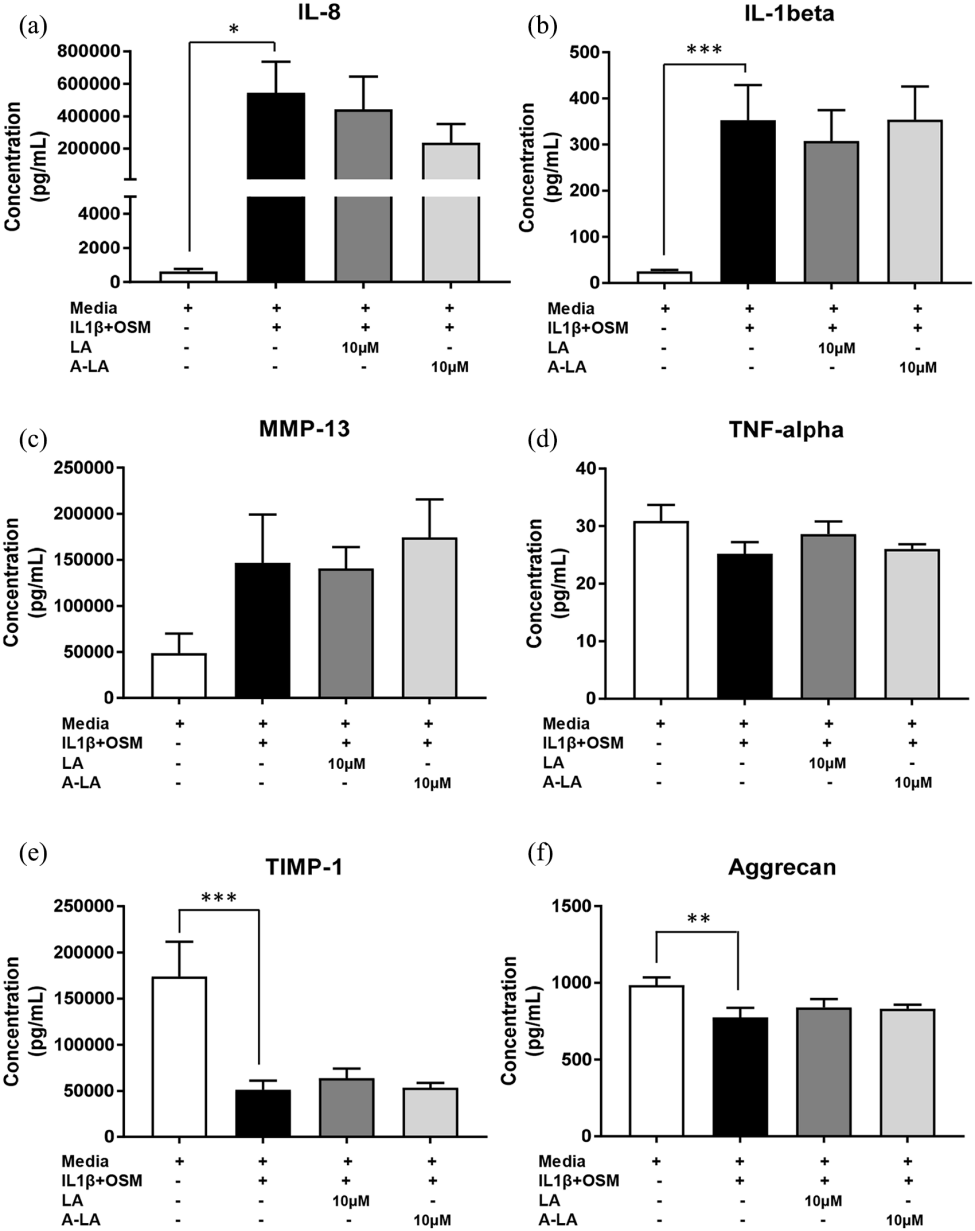
Effect of LA and A-LA on modulation of cytokines and cartilage-related proteins in human OA cartilage explants. Cartilage explants were stimulated for 14 days, supernatants collected and protein concentrations were determined by Luminex technology. (a) IL-8, (b) IL-1β, (c) MMP-13, (d) TNF-α, (e) TIMP-1 and (f) aggrecan. Results are shown as mean ± SEM of triplicate determinations per sample (n = 6). *p ⩽ 0.05, **p ⩽ 0.01, *p ⩽ 0.001 versus control media.

We subsequently investigated the effect of both LA and A-LA on protein levels in supernatants from cartilage explants on day 14 post stimulation. Despite the positive results obtained by immunohistochemistry, no significant differences were observed in supernatant levels of pro-inflammatory mediators IL-8, IL-1β, MMP-13, TNF-α, or in levels of TIMP-1 and aggrecan (Figure 5(a)–(f)). Culture with IL-1β and OSM induced a large and significant increase in IL-8 and IL-1β production (Figure 5(a) and (b)) and a significant decrease in TIMP-1 and aggrecan (Figure 5(e) and (f)). Levels of IL-8 were reduced not only with A-LA (235,934 ± 115,733 vs 544,450 ± 191,948, *p* = 0.262) (Figure 5(a)) but also with LA (442,021 ± 203,050 vs 544,450 ± 191,948, *p* = 0.262) (Figure 5(a)), although this did not reach statistical significance.

## Discussion

Traditionally OA has not been considered to be an inflammatory form of arthritis; however, there is increasing evidence to suggest that inflammation is involved in OA development and progression and, furthermore, that chondrocytes directly respond to inflammatory cues mounting a response via NF-κB leading to MMPs, COX, chemokine and cytokine expression.^17^ A Mediterranean diet, which is rich in olive oil, is believed to confer various health benefits, a number of which^31^ appear to overlap with those attributed to non-steroidal anti-inflammatory drugs. For example, oleocanthal acts as a natural anti-inflammatory compound that has a potency and profile strikingly similar to that of ibuprofen. Although structurally dissimilar, both these molecules inhibit the same cyclooxygenase enzymes in the prostaglandin-biosynthesis pathway.^32^ Recently, accumulating experimental, clinical and epidemiological data have provided support for the traditional belief of the beneficial effect afforded by olive derivatives.^33–35^

Our results demonstrate that the nutraceutical LA and, especially the acetylated derivative A-LA, display an anti-inflammatory profile and reduce the cartilage degradation observed in OA pathology. The upregulation of NO in chondrocytes has been seen to modulate cytokine expression, suppress synthesis of matrix collagen and PG, suppress energy metabolism and activate MMPs^36^ thereby contributing to OA physiology. The end products of NO metabolism can cause cell death via DNA damage, protein degradation and lipid peroxidation.^37^ Elevated nitrites production has been observed in OA serum and synovial fluids^29^ and, our results correlate with previous studies^38,39^ indicating elevated nitrite is present in the supernatants from cartilage explants when stimulated with IL-1β and OSM, corresponding to the increase in *NOS2* expression. This was also evident in the positive control samples in the current immunohistochemical studies with positively stained chondrocytes observed throughout the superficial and deep cartilage layers. Treatment with nutraceuticals from EVOO reduced the levels of nitrites, correlating with similar reductions seen in *NOS2* gene expression, with, in particular, A-LA reducing nitrites and *NOS2* expression.

Safranin-O and Alcian blue/Sirius red staining of the positive OA controls showed obvious losses in PG from the superficial layers with only discrete stained regions present in the deep zones. Interestingly, Sirius red only stains types I and II collagen following PG loss (as otherwise the stain is prevented from reaching the collagen).^30^ We demonstrate PG could be recovered upon treatment with A-LA. Furthermore, the data from the s-GAG release studies demonstrate that the treatment with both nutraceuticals reduced PG loss when compared to the positive control. The PG loss in the cartilage samples was observed to correlates with the data observed for MMP13. The expression of this MMP, also known as collagenase 3, is typically restricted to connective tissues compared to other MMPs.^40^ MMP13 enhanced activity has been observed in cartilage degradation in OA^41^ as MMP13 degrades type II, IV and IX collagens, small PGs, perlecans and osteonectin^42^ driving the erosion of the cartilage collagen network. Furthermore, mouse models have demonstrated that cartilage-specific deletions of MMP13 decelerated OA progression.^40^ Interestingly, articular chondrocytes express low basal levels of *MMP13*, however, inflammatory signalling induces enhanced activity. The immunohistochemical data in the current study confirmed *MMP13* upregulation in the positive control, particularly, in the superficial layer as previously seen,^42^ as well as gene expression and, both indices were reduced following A-LA treatment. A previous study with other nutraceuticals demonstrated that the fatty acid, eicosapentaenoic acid (EPA) and docosahexaenoic acid (DHA) reduced mRNA and protein levels of MMP-13 in bovine chondrocytes.^43^ In a previous study, oleocanthal inhibited catabolic and inflammatory mediators in LPS-activated human primary OA chondrocytes through the MAPKs/NF-κB pathways.^18^ The nutraceuticals analysed in this current study did not decrease the different cytokine levels in culture medium, in contrast to the results reported in a previous study^44^ which may reflect experimental differences, given in the present study, the selected time was day 14 and thus more time points should be analysed in future studies. Furthermore, Szychlinska and colleagues showed that a combination of physical activity and a Mediterranean diet has a protective effect not only on early OA but also on muscle atrophy and hepatic steatosis.^19^ While information on LA bioactivity is limited, LA has been shown to behave as an antioxidant^26^ and, to induce apoptotic cell death in HER2-dependent breast cancer, showing moderate cytotoxicity against a panel of human cancer cell lines.^45^ Antimigratory activity of LA against the highly metastatic human breast cancer cell line MDA-MB231 has also been assessed^46^; and more recently, LA has been reported to display anti-proliferative effects in liver cancer cell lines^47^ and mitochondrial dysfunction in models of early Alzheimer’s disease and brain ageing.^48^

**Figure 6.**
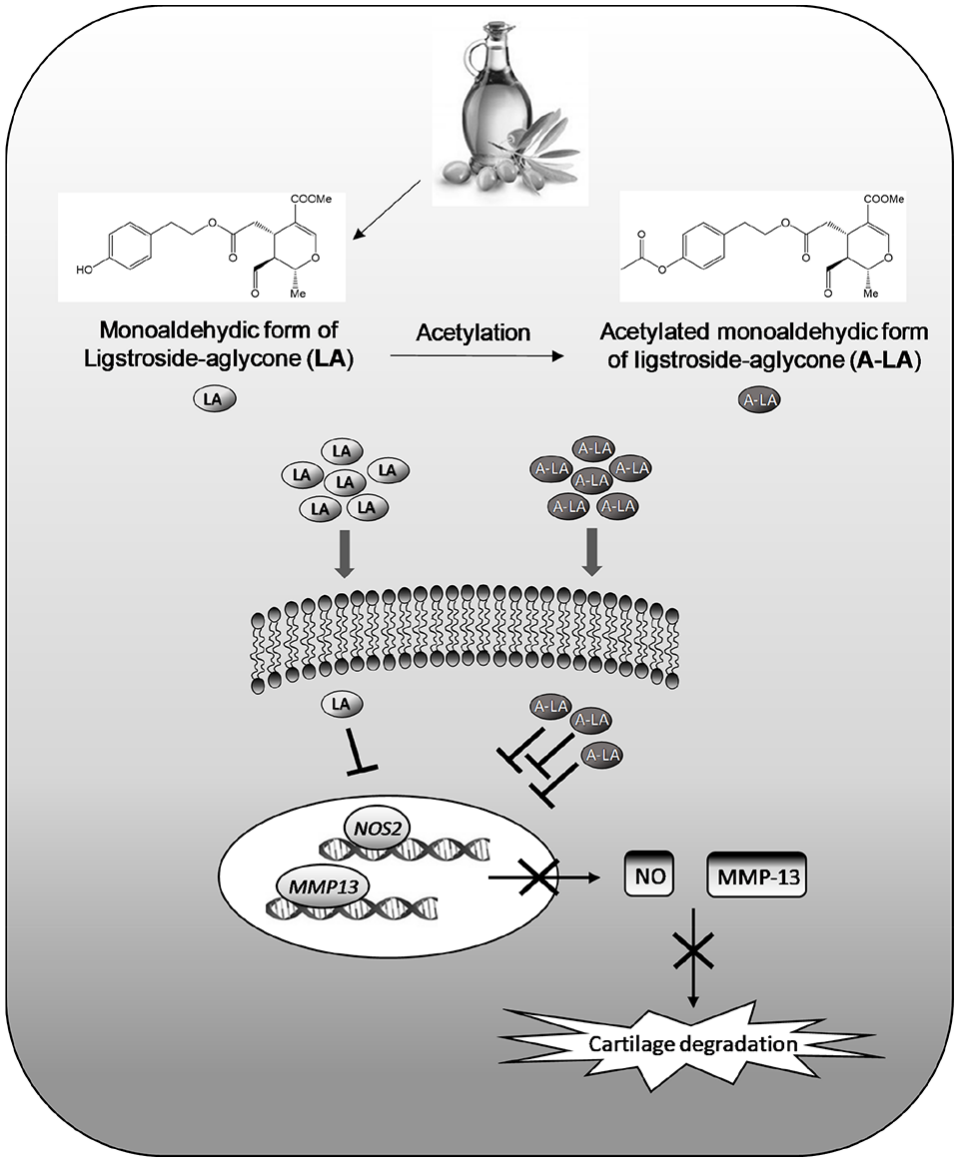
Mechanism of action of nutraceuticals LA and A-LA in human primary chondrocytes. Nutraceuticals alleviate inflammation and cartilage degradation inhibiting the transcription of key catabolic genes in OA pathology (e.g. NOS2 and MMP-13). Acetylated compounds display an improved pharmacokinetic/pharmacodynamics profile related to the modification of the chemical structure that may favour penetration through the cell membrane.

Studies have shown the importance of acetylated derivatives of natural phenols given their lipophilic nature facilitates transfer across the cytoplasmic cell membrane and cell uptake, offering a possible protection of membrane components.^49^ Acetylation of polyphenols may confer improved properties to these molecules, including enhanced bioavailability, cell membrane penetration and enhanced anti-inflammatory and antioxidant activities, among others.^50,51^ These findings are in line with previous in vitro studies carried out with other olive phenols, including oleuropein,^49^ hydroxytyrosol and peracetylated hydroxytyrosol,^52^ hydroxytyrosol acetate and its derivatives^53^; with better hydrophilic/lipophilic balance, these compounds exhibited strong anti-inflammatory effects in LPS-stimulated murine isolated peritoneal macrophages. It is possible that the acetylation of aliphatic hydroxyl groups in LA results in an increase in the lipophilic profile of LA, explaining the reduction in cartilage degradation exhibited after derivatives tested in comparison with their natural pattern, LA. We believe, this is the first time that such a phenomenon has been shown in OA (Figure 6).

A number of studies carried out by us and others have shown a pivotal role for epigenetics in OA.^29,30,54–57^ OA is characterised by the aberrant expression of catabolic genes, including aggrecanases, *iNOS* and prostaglandins due to hypomethylation at CpG sites which may be itself driven by inflammatory cytokines.^29^ For NOS2, it remains unclear if this arises as a consequence of a reversal in the epigenetic status within the *NOS2* gene itself in OA chondrocytes or, if the observed effects with the nutraceuticals were as a consequence of NF-κB, as has previously been observed.^12,58^ Analysis of the methylation status of the DNA from the cartilage will contribute to explaining the exact effects A-LA has on catabolic gene expression. Thus, epigenetic processes appear to contribute significantly to the development of OA and lead to the hypothesis that nutraceutical compounds could be implemented therapeutically given their anti-inflammatory effects that may proceed by reversing epigenetic changes that occur in OA. There is indeed a wealth of literature linking diet with epigenetic regulation.^59–62^ Dietary changes play a significant role in modulation of the epigenome, including DNA methylation, post-translational histone modifications and noncoding RNAs. Diet-induced epigenetic changes may be transmitted to the following unexposed generation through the germline. Thus, such diet-induced epigenetic changes may provide the key mechanistic link between diet-induced and thus, may serve as targets of intervention.^63^ The effect of dietary components on epigenetic imprinting, exploring alternative nutrition-based therapeutic approaches and developing tools for personalised diet may improve health and increase life expectancy.^64^

The limitations of this study include the absence of methylation studies, with preliminary studies inconclusive given the limited quality DNA available from the OA cartilage explants. It is anticipated that other epigenetic mechanisms could be regulated by these compounds, including miRNAs or histones marks. Potentially, the acetylated compound A-LA could transfer an acetyl group from the phenolic hydroxyl to the amino group of the lysine residue in the N-terminal tail of histones.

In summary, A-LA appears a promising nutraceutical for the evaluation of the treatment of OA. This new synthetic LA derivative exhibits an enhanced anti-inflammatory profile that of the natural compound LA, potentially as a consequence of improved pharmacokinetic/pharmacodynamics profile related to the modification of the chemical structure. Future work will seek to determine the pathways affected by A-LA such as the location of epigenetic alterations, which will offer new approaches/information on optimising dosage and delivery and strategies for designing a therapeutic intervention for the treatment of OA. In summary, dietary supplements, such as A-LA in particular, could represent a new strategy for OA prevention, particularly in the early stages of the disease with significant therapeutic implications for an aging demographic.
